# Linkage disequilibrium reveals different demographic history in egg laying chickens

**DOI:** 10.1186/1471-2156-11-103

**Published:** 2010-11-15

**Authors:** Saber Qanbari, Maria Hansen, Steffen Weigend, Rudolf Preisinger, Henner Simianer

**Affiliations:** 1 Animal Breeding and Genetics Group, Department of Animal Sciences, Georg-August University, 37075 Göttingen, Germany; 2 Institute of Farm Animal Genetics, Friedrich-Loeffler-Institut, Neustadt-Mariensee, Germany; 3 Lohmann Tierzucht GmbH, Cuxhaven, Germany

## Abstract

**Background:**

The availability of larger-scale SNP data sets in the chicken genome allows to achieve a higher resolution of the pattern of linkage disequilibrium (LD). In this study, 36 k and 57 k genotypes from two independent genotyping chips were used to systematically characterize genome-wide extent and structure of LD in the genome of four chicken populations. In total, we analyzed genotypes of 454 animals from two commercial and two experimental populations of white and brown layers which allows to some extent a generalization of the results.

**Results:**

The number of usable SNPs in this study was 19 k to 37 k in brown layers and 8 k to 19 k in white layers. Our analyzes showed a large difference of LD between the lines of white and brown layers. A mean value of *r^2 ^*= 0.73 ± 0.36 was observed in pair-wise distances of < 25 Kb for commercial white layers, and it dropped to 0.60 ± 0.38 with distances of 75 to 120 Kb, the interval which includes the average inter-marker space in this line. In contrast, an overall mean value of *r^2^= *0.32 ± 0.33 was observed for SNPs less than 25 Kb apart from each other and dropped to 0.21 ± 0.26 at a distance of 100 kb in commercial brown layers. There was a remarkable similarity of the LD patterns among the two populations of white layers. The same was true for the two populations of brown layers, while the LD pattern between white and brown layers was clearly different. Inferring the population demographic history from LD data resulted in a larger effective population size in brown than white populations, reflecting less inbreeding among brown compared to white egg layers.

**Conclusions:**

We report comprehensive LD map statistics for the genome of egg laying chickens with an up to 3 times higher resolution compared to the maps available so far. The results were found to be consistent between analyzes based on the parallel SNP chips and across different populations (commercial vs. experimental) within the brown and the white layers. It is concluded that the current density of usable markers in this study is sufficient for association mapping and the implementation of genomic selection in these populations to achieve a similar accuracy as in implementations of association mapping and genomic selection in mammalian farm animals.

## Background

Linkage disequilibrium (LD), the non-random association of alleles at two or more loci, has been in the focus of much attention recently, because of its usefulness in determining the actual genes responsible for variation of economically important traits through association mapping in livestock populations [1,2]. Information on the extent of LD in genomic regions harboring quantitative trait loci (QTLs) is necessary for effective applications of marker assisted selection in commercial breeding programs [3]. Another appealing application of LD information is inferring population demography based on the changes in the historical effective population size (*Ne*). Theoretical analyzes based on the well-known formula suggested by Sved [4] allow assessing the development of historical effective population size by comparing the decay of LD over intervals of increasing map distance. However, additional factors such as genetic drift, selection within populations, and population admixture can also cause LD between marker pairs or markers and traits, and these mechanisms might affect non-syntenic loci across chromosomes, i.e. in the absence of physical linkage. Therefore, information on the local magnitude of LD and a detailed profile of the recombination variability and blocking structure is of key importance for the genome-based analysis of population history and for the fine-tuning of applications like association mapping and genomic selection.

Since the first genetic linkage map of chicken was published by Serebrovsky and Petrov [5] several versions of linkage maps, mostly based on microsatellite markers, have been constructed [6-8]. These studies have reported substantial LD over long distances for pairs of loci being up to 5 cM apart. As in many other species, microsatellites recently have been replaced by single nucleotide polymorphisms (SNPs) and maps based on high-density SNP arrays reveal much lower levels of LD, limited to ≤ 100 kb [9-11]. The latest map included 13,340 markers with a total map length of 3,054 cM in broilers [12].

The availability of large-scale SNP data sets in the chicken genome [13] allowed to increase the marker density and to achieve a comprehensive coverage of the chicken genome. Benefiting from recently established Illumina 60 K genotyping chips for the chicken genome, in this study, we (1) construct a new LD map with higher resolution, (2) compare the extent and structure of LD and haplotype blocks between lines of brown and white egg laying chickens, and (3) use LD statistics to estimate and compare present and historic effective population sizes of the studied populations which have a diverse historical background. Since these analyzes are based on the genotypes available from two independent SNP chips and two independent brown and white layer lines, respectively, the results can be validated across genotyping technologies and populations.

## Methods

### Populations studied

This study is based on two experimental (E) and two commercial (C) populations, each comprising a white (W) and a brown (B) layer breed (Table 1). We henceforth use the code WE for the experimental white layer line, and accordingly BE, WC, and BC. Experimental and commercial lines with systematical differences in breeding history, sample size, and the SNP chip were used for genotyping. This structure allows to some extent a generalization of results.

**Table 1:** Description of the genotyped birds.

Dataset	Line	Breed	Purpose	Animals (n)	Breeding scheme	Pedigree
I	WE	White Leghorn	experimental	25	conservation breeding using rooster rotation	2000-2007
I	BE	New Hampshire	experimental	25		
		
II	WC	White Leghorn	commercial	200	commercial selection programme	1992-2008
			
II	BC	Rhode Island Red and White Rock	commercial	204		
				∑ 454		

Table 1 summarizes information concerning population, sample size, status and the main breeding purpose and scheme of the 454 birds composing two data sets of this study.

**Data set I **comprised two experimental pure bred chicken lines, WE and BE representing White Leghorn and New Hampshire chickens, respectively. The selected lines have been maintained at the Institute of Farm Animal Genetics (FLI) Neustadt, Mariensee. Hatching eggs for White Leghorn chicken were imported to former Institute of Small Animal Breeding in Celle in 1965 from the Cornell Line K selected for resistance to neoplasm. The sub-line WE had been established with the aim of selecting for susceptibility for ALV A/B infection. BE is an experimental brown layer chicken line founded in 1970 in the former German Democratic Republic (VEG Vogelsang). Each population was composed of 10 sire families made up by 1 sire and 10 dams per family. After each generation roosters were systematically rotated among families (e.g; a rooster from first family artificially inseminates second family and so on) with no selection and a generation interval of 1 year (for more information see [14]).

Twenty five birds of each experimental line were sampled from two consecutive years (2003 and 2004), reflecting two subsequent generations. In each line the sire of each family (e.g., 10 sires per line) and one or two females per family (n = 15), respectively, were sampled to represent the complete variation of the lines.

**Data set II **was composed of two commercial egg laying strains of the Lohmann Tierzucht GmbH. The first population BC comprised 204 laying hens produced from a two way cross of Lohmann Brown lines. The pure lines have their origins in Rhode Island Red and White Rock, respectively, and are closed populations which are selected for all economically important traits under pure and crossbred conditions using genetic evaluations based on multiple trait best linear unbiased prediction. The genotyped BC individuals are F1 animals of a cross of pure breeds. This means, that after haplotyping we observe in one individual for each autosome a combination of two chromosomes originating from either of the two parental breeds, respectively, without being able to assign haplotypes to the breed of origin. Note, however, that no recombination across haplotypes of the two different parental lines was possible and hence the observed LD was not generated by population admixture. The parameters estimated from these genotypes thus reflect a mixture of two purebred brown layer populations, rather than a single population.

The second commercial population WC consisted of 200 White Leghorn females from a pure line. The samples comprised were half sib groups. It is generally recommended to use maternal haplotypes for evaluating the extent and pattern of LD, because LD from paternal haplotypes may reflect LD within sire families, rather than in the wider population. However, these relationships are not expected to bias estimates of LD, because the small size of half sib families limits genetic contribution of each sire in the relatively large sample taken from this line [9]. Sampling from both commercial lines was done in 2008.

### SNP genotypes and data preparation

DNA was extracted from fresh blood samples using standard DNA isolation procedures [15]. SNP genotyping for data set I was done by DNA LandMarks Inc., Quebec, Canada, using publicly available chicken 60 K chips produced by Illumina Inc. for the genome-wide marker-assisted selection (GWMAS) Consortium. The total number of SNPs and the mean distance between adjacent markers in this chip were 57'635 and 17.91 kb, respectively.

Data set II was genotyped with a new chicken genotyping BeadChip which was ordered exclusively by Lohmann Tierzucht GmbH and established in parallel by Illumina Inc. The Lohmann chip contains a total of 36'455 SNPs with a mean neighbor marker distance of 29.82 kb.

For the purposes of this study, only autosomal SNPs were included in the LD analysis. To ensure the highest possible data quality, a series of filters was employed to remove lower quality markers and insecure genotypes of individuals. We eliminated samples with ≥ 5% missing genotypes and SNPs which were assigned to unmapped contigs or were not positioned according to the latest reference assembly of the chicken genome (Build 2.1). Genotypes were discarded if they had quality scores < 95%. We also restricted the analysis to SNPs showing a minor allele frequency (MAF) of at least 5% after filtering. SNPs not matching this criterion were excluded for two reasons: it has been shown that SNPs with low frequency have little power for the detection of LD [16,17]. Furthermore, SNPs with lower allele frequencies increase the number of lower-frequency haplotypes, and the inclusion of rare population-specific SNPs leads to the addition of population-specific haplotypes [18]. The number of heterozygous loci was determined and used to estimate the average heterozygosity for all individuals. Furthermore, MAF and observed heterozygosity were determined for each SNP.

### Haplotype inference and block partitioning

Haplotypes generally have more information content than individual SNPs in genome-wide studies, and they provide valuable information on the evolutionary history of a population. In this study, the inference of haplotype pairs as well as the imputation of missing genotypes was carried out directly on the basis of unphased genotype data for each chromosome within each population using the EM algorithm implemented in fastPHASE [19].

Genomic haplotypes can be partitioned into discrete blocks in such a way that haplotype diversity is constrained within each block and is high between the blocks. We used HAPLOVIEW v4.1 [20] to determine haplotype block boundaries and to estimate within-block haplotype diversity. The algorithm suggested by Gabriel *et al. *[21] was used to determine the blocking structure by defining a pair of SNPs to be in ''strong LD'' if the upper 95% confidence bound of D' is between 0.7 and 0.98.

### Measure of LD

Several statistics have been used to measure the LD between a pair of loci. We used *r*^2 ^which is generally accepted as a robust LD parameter [22-24] with direct relevance for implementations related to association mapping and genomic breeding value estimation.

Consider 2 loci, A and B, each locus having 2 alleles (denoted A_1_, A_2_; B_1_, B_2_, respectively). We denote *f*_11_, *f*_12_, *f*_21_, and *f*_22 _as the frequencies of the haplotypes A_1_B_1_, A_1_B_2_, A_2_B_1_, and A_2_B_2_, respectively; *f*_*A*1_, *f*_*A2 *_*f*_*B*1_, and *f*_*B*2 _are the frequencies of A_1_, A_2_, B_1_, and B_2_, respectively. Following Hill and Weir [25],

$$r^{2}=\frac{{\left(f_{11}f_{22}-f_{12}f_{21}\right)}^{2}}{f_{A1}f_{A2}f_{B1}f_{B2}}$$

### Estimation of historical effective population size

*N_e _*can be estimated from LD data and the availability of dense markers has made this option feasible. For autosomal loci assuming a linear population growth, the expected *r*^2 ^between neutral markers can be related to genetic effective population size *N*e and genetic distance *c *(in Morgan units) according to the formula

$$E(r^{2}=\frac{1}{\kappa +4Ne\times c}+\frac{1}{n})$$

where *k *= 1(≈ 2) if mutation is (not) taken into account [4,26] and *n *is the chromosomal sample size. Simulation studies revealed that estimates of past effective population sizes are not greatly affected by departure from the assumption of a linear population growth [27]. Therefore, without considering mutation (*κ *= 1)in the model, the effective population size *Ne*, in $\frac{1}{2c}$ generations ago, can then be estimated from observed *r*^2 ^values related to a given genetic distance *c*[27,28].

While in most mammals the variability of the average ratio of genetic vs. physical distance across chromosomes is small [29,30], an approximately eightfold (2.5 to 21 cM/Mb) variation in recombination rate was found among chicken chromosomes [31], with a much higher recombination rate per centiMorgan on the microchromosomes compared to the macrochromosomes. To account for this fact we calculated for a marker interval of physical length *x_i _*(in kilobasepairs) on chromosome *i *the corresponding genetic length of the interval to be $c_{i}={\bar{\rho }}_{i}x_{i}$, where ${\bar{\rho }}_{i}$ is the average ratio of Morgan per kilobasepair on chromosome *i*. The values of ${\bar{\rho }}_{i}$ were taken from the genetic and physical lengths of chromosomes as reported by ICGSC [31]. The decay of *Ne *was then analyzed for each population using LD values averaged in bins of linkage distance and inferring the changes in effective population size from nearly 800 generations ago.

## Results

### Marker statistics

Table 2 presents a descriptive summary of SNP numbers and frequencies, genome coverage and inter-marker distance for each population. As shown a substantial proportion of the genetic variation represented by either of the SNP chips used is not displayed in any of the four selected lines. Clearly, the magnitude of the fixation is greater in white layers (58 and 62% in WE and WC, respectively) compared to the brown layers (27 and 32% in BE and BC, respectively). Line BE with 37'075 SNPs in use having 27.8 Kb inter-marker distance and WC with 8'447 SNPs in use and 112.3 Kb average adjacent marker spacing showed the densest and sparsest marker panels in this study.

**Table 2:** Characteristics of marker panels used in different populations.

	Dataset I		Dataset II	
	**BE**	**WE**	**BC**	**WC**

**Total Number of SNPs**	57'635	57'635	36'455	36'455
# SNPs monomorph	15'675	33'915	11'800	22'815
# SNPs ungenotyped	836	894	982	1'059
# SNPs with MAF < 5%	2'893	1'883	2'392	450
Genome coverage^1 ^(Mb)	1031	1027	956	949
# SNPs in use	37'075	19'802	19'892	8'447
# common SNPs^2^	14653		6072	
Mean adjacent marker spacing (kb)	27.8	51.8	48.0	112.3
Mean MAF	0.28 ± 0.13	0.34 ± 0.14	0.28 ± 0.21	0.24 ± 0.15
Mean observed heterozygosity	0.35 ± 0.15	0.34 ± 0.15	0.47 ± 0.21	0.37 ± 0.13
Mean expected heterozygosity	0.37 ± 0.12	0.36 ± 0.12	0.37^3 ^± 0.12	0.36 ± 0.12

^1^Genome coverage after filtering

^2^Number of common SNPs in use between BE and WE, and between BC and WC

^3^Expected heterozygosity under the assumption that BC was a pure line, which is not the case

**Figure 1:**
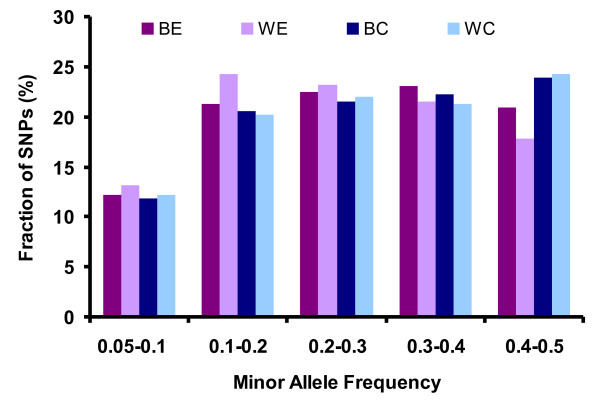
**Distribution of minor allele frequency of SNPs in populations**.

Figure 1 displays the distribution of MAF in the populations studied. Nearly 70% of SNPs in four populations had MAF larger than 0.2, implying that the effect of low MAF on the overall LD estimates should be small. The almost uniform distribution across frequency classes is due to the ascertainment bias, as discussed by Muir et al. [32] and presumably can be explained by the optimization of SNP arrays with respect to a uniform SNP spacing and MAF distribution. The heterozygosity on average was estimated higher for the brown vs. the white layers and for the commercial vs. the experimental populations. Note that observed heterozygosity is excessive in line BC because the genotyped animals are F1 crosses of two pure lines; hence the degree of heterozygosity is partly reflecting the genetic distance between those lines.

### Comparison of haploblock structure

**Figure 2:**
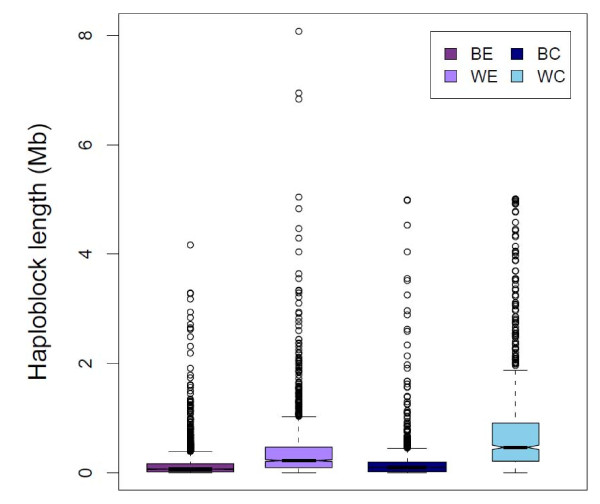
**Box plot of haploblock size in different populations**.

Table 3 and Figure 2 present the statistics of the genome wide haploblock distribution across the populations studied. On average about 80% of the markers formed haplotypic tracts in WL, while this ratio was between 40% and 50% for BL. While line WC had 741 blocks spanning over 50% of the genome, line BE had 2562 blocks covering only 35% of the genome, which reflected the longest and shortest blocking structures in the genome, repectively. The mean number of SNPs in the haplotypic tracts ranged from 4.4 to 9.7 among populations with a maximum of 83 SNPs assembling a block in WE.

**Table 3:** A summary statistics of haploblock structure across the populations.

	Dataset I		Dataset II	
	**BE**	**WE**	**BC**	**WC**

Blocks (n)	2562	1519	2088	741
Genome Coverage (Mbp)	373.7	599.4	337.1	592.0
Mean Block Length (kb)	145.8 ± 280.9	394.6 ± 605	161.4 ± 324.4	798.9 ± 1001.6
BSNPs^1 ^(%)	41.8	74.3	46.6	85.3
Mean nBSNPs^2^	6.1 ± 6.00	9.7 ± 9.3	4.4 ± 3.7	9.7 ± 8.6
Max nBSNPs^3^	70	83	69	57

^1 ^The total frequency of SNPs forming haploblocks

^2, 3 ^the mean and maximum number of SNPs forming haploblocks, respectively

### Comparison of the extent of LD across the genome

We examined the extent of LD in each line separately. Table 4 summarizes the LD as a function of genetic distance for the lines studied. Analysis of LD showed a large difference between lines representing white and brown layers in both data sets. A mean value of *r^2 ^*= 0.73 ± 0.36 was observed in pair-wise distances of <25 Kb for WC which dropped to *r^2 ^*= 0.60 ± 0.38 at distances from 75 to 120 Kb, the interval which includes the average inter-marker space in this line. In line BC an overall mean value of *r^2^= *0.32 ± 0.33 was observed for SNPs less than 25 Kb apart from each other, which dropped to 0.21 ± 0.26 at distances from 75 to 120 Kb. There was a remarkable similarity of the LD patterns in two populations of WL from data sets I and II.

**Table 4:** Comparison of the strength of LD versus physical distance.

	Data set I				Data set II			
	**BE**		**WE**		**BC**		**WC**	

**Distance (Mb)**	**Mean ± SD**	**Useful LD (%)**	**Mean ± SD**	**Useful LD (%)**	**Mean ± SD**	**Useful LD (%)**	**Mean ± SD**	**Useful LD (%)**

< 0.025	0.37 ± 0.34	48.6	0.66 ± 0.38	76	0.32 ± 0.33	36.3	0.73 ± 0.36	79
0.025-0.05	0.32 ± 0.32	43.3	0.61 ± 0.39	73	0.28 ± 0.30	31.4	0.67 ± 0.38	74
0.05-0.075	0.29 ± 0.30	39.9	0.56 ± 0.39	68	0.24 ± 0.28	27.9	0.64 ± 0.38	72
0.075-0.12	0.27 ± 0.29	36.7	0.51 ± 0.38	64	0.21 ± 0.26	24.5	0.60 ± 0.38	68
0.12-0.2	0.24 ± 0.26	32.8	0.45 ± 0.37	59	0.18 ± 0.23	19.8	0.54 ± 0.38	63
0.2-0.5	0.19 ± 0.23	25.0	0.35 ± 0.33	47	0.13 ± 0.18	12.9	0.44 ± 0.36	52
0.5-1.5	0.12 ± 0.17	13.8	0.19 ± 0.24	25	0.07 ± 0.12	4.8	0.26 ± 0.30	31
1.5-3	0.07 ± 0.12	6.5	0.09 ± 0.15	10	0.04 ± 0.07	1.5	0.15 ± 0.23	16
3-5	0.05 ± 0.08	3.4	0.05 ± 0.09	04	0.02 ± 0.04	0.4	0.08 ± 0.15	6
5-10	0.04 ± 0.06	1.5	0.04 ± 0.05	01	0.01 ± 0.02	0.1	0.03 ± 0.06	1

The mean *r*^2 ^values and the proportion of SNP pairs that shows statistically significant LD for markers apart up to 10 Mbp are presented for the all populations.

**Figure 3:**
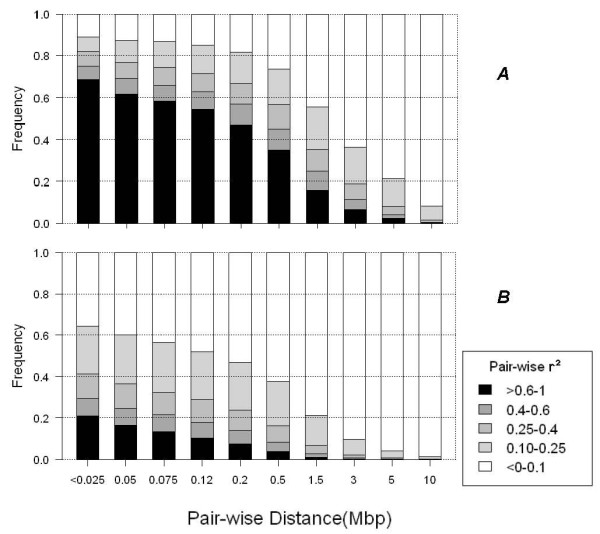
**Comparison of the fraction of marker pairs with different *r*^2 ^levels (< 0.1, 0.1-0.25, 0.25-0.4, 0.4-0.6 and >0.6, depicted by different colors) in different distance bins between commercial WC (A) and BC (B) lines**.

In order to visualize the decay of LD we stacked *r*^2 ^and plotted them as a function of inter-marker distance categories (< 0.025, 0.025-0.05, 0.05-0.075, 0.075-0.12, 0.12-0.2, 0.2-0.5, 0.5-1.5, 1.5-3, 3-5 and 5-10 (Mbp) (Figure 3). This genome-wide bar plot illustrates the rate at which LD decays with physical distance and forms the basis for comparison between studies. The decay of LD showed a clear exponential trend with physical distance which is typically found in all other data sets and agrees with previous results [[8,32,33] and [11]] and with theory [4].

The threshold for LD being useful for association studies was chosen to be 0.3 in accordance with other studies [34,23]. The average proportion of SNPs in useful LD for White and Brown layer lines for the distance of <25 Kb. was 77.5% and 42.5%, respectively, This proportion drops to 61% and 26.3%, respectively, for SNPs 100 Kb apart from each other. These results showed that the useful LD extended over 5 and 2 Mbp in White and Brown layers, respectively, so that the proportion of SNP pairs in useful LD is above 5%.

**Figure 4:**
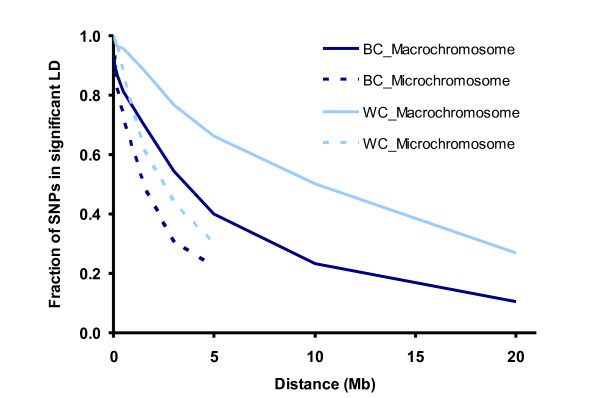
**The proportion of SNP pairs in significant (P ≤ 0.05) LD plotted against the physical distance for macro- and microchromosomes in the commercial lines**.

To get insight into the significance of LD in different bins of inter-marker distance, we tested the level of departure from expected haplotype frequencies under linkage equilibrium (LE) between markers up to 20 Mb apart using a χ^2^-test (Foulkes, 2009). The proportion of SNP pairs deviating significantly (P ≤ 0.05) from LE was then computed and compared between micro- and macrochromosomes within commercial populations (Figure 4). As expected, a considerable difference in proportion of SNP pairs in significant LD was observed between brown and white layers. In white layers 27% of marker pairs showed significant departures at distances up to 20 Mb, while this proportion dropped to 10% in brown layers. A substantial difference was also observed between macro-versus microchromosome which can be attributed to the much higher recombination rates on short chromosomes. It should be noted, that for intervals of 3 to 5 Mb between 30 and 70% of SNP pairs are in significant LD, despite the fact that the average *r^2 ^*is only between 0.02 and 0.08 (Table 2).

### Genome-wide variation in LD

**Figure 5:**
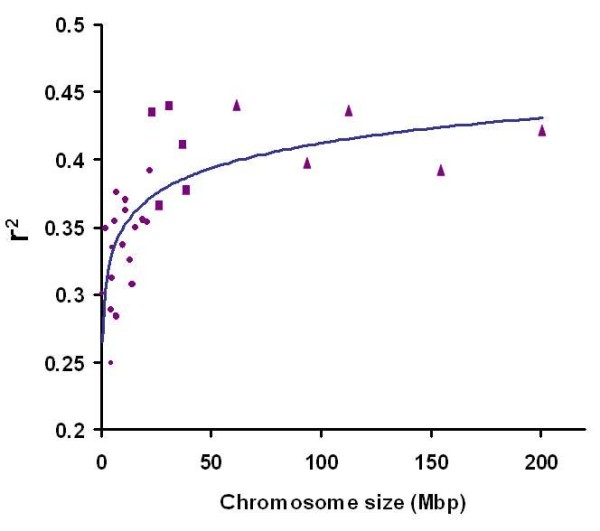
**This plot depicts the relationship between the strength of LD versus length of the chromosomes in experimental Brown laying hens**. Triangles, squares and circles are presenting mean *r^2 ^*across macro, intermediate and microchromosomes, respectively. The regression line was fitted as y = 0.0269Ln(x) + 0.2881 with R^2^= 0.52.

The extreme heterogeneity of chromosome size is a specific feature of avian genomes such that the genome is organized into a few very large chromosomes and many very small chromosomes with substantial structural differences between them. However, there is not a unique classification for chicken chromosomes size. Some reports have classified them as 8 pairs of macrochromosomes, one pair of sex chromosomes, with the remaining 32 pairs being intermediate or microchromosomes [35]. Other arrangements such as the one used by the International Chicken Genome Sequencing Consortium include five pairs of macrochromosomes, five pairs of intermediate chromosomes, and twenty-eight pairs of microchromosomes [7,36]. Recombination rates have been found to differ between micro- and macrochromosomes in chickens. This variability first was identified in the chicken genome reporting 2.8-6.4 cM/Mb [31]. For illustration we considered the extent of LD, represented by the average *r^2 ^*for a given physical segment length across chromosomes. Figure 5 shows this statistic (*r^2 ^*for an interval size of up to 25 Kb) as a function of the physical chromosome length. We fitted a logarithmic function, giving an acceptable fit with an R^2 ^value of 0.52. It should be noted that the often used classification in macro- and microchromosomes [31] does not hold well under this perspective, since the functional relationship between chromosome length and extent of LD is continuous rather than di- or trichotomous.

### Inferring population demographic history from LD

**Figure 6:**
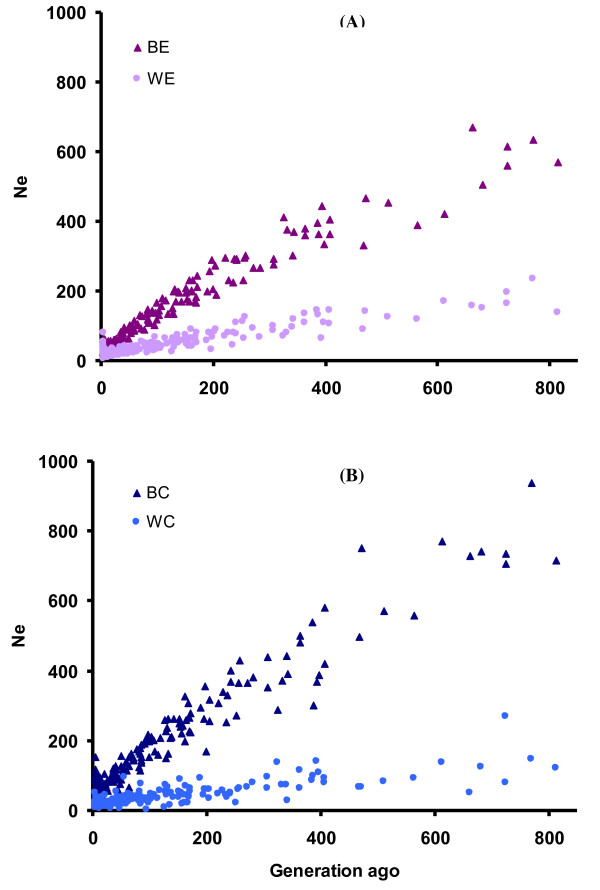
**Decay in effective population size over generations for experimental (A) and commercial lines (B) estimated from linkage disequilibrium data**.

Former reports have shown ensuing reduction in effective population size for commercial breeding populations caused by the intensive artificial selection for many generations [[33,9] and [11]]. In this study we fitted Sved's [4] equation to the *r^2 ^*values from each line with sex-averaged genetic distances available for each chromosome [31]. *Ne *estimates from experimental populations were corrected for chromosomal sample size. In general, the *Ne *size was larger in BL than WL populations based on the comparison of two data sets and parallel SNP panels reflecting less inbreeding among brown, than white, egg layers (Figure 6). The recent (5 generations ago) and ancestral (200 generations ago) effective population size was estimated to be less than 70 and 300, respectively, for BL versus 50 and 100 individuals, respectively, for WL, displaying clear evidence of a decline in effective population size.

We have further delineated the influence of demography on LD by contrasting the differences observed between the WL and BL populations in different data sets. The commercial and experimental lines both revealed a clear evidence of decaying *Ne*, however, the trend of decaying ancestral *Ne *appears to be stronger in the commercial populations than in the experimental lines. In contrast recent *Ne *for commercial lines is in general larger than for experimental lines, reflecting different constraints and breeding management in their more recent history. The latter confirms the estimates of the current *Ne *based on information from pedigrees of experimental lines (R22, L68) in comparison to the Lohmann commercial lines of LSL-A and LB-A [37].

## Discussion

In this article we report the construction of a whole-genome LD map using genotypes from white and brown egg laying chickens. We analyzed data from two parallel SNP chips comprising more than 40'000 genetic markers, covering 28 chromosomes but leaving other micro-chromosomes uncovered because they had not sufficiently large numbers of SNPs to obtain accurate results. This is so far the most comprehensive study of LD with high density SNP panels in breeding lines of egg laying chickens and currently reflects the best estimates of genome-wide LD based on the number of animals screened and number of SNPs genotyped.

We found a widespread LD among markers separated up to 10 Mb apart. Although there were significant differences in the degree of LD between lines, overall LD levels were high. The magnitude of LD appeared to be greater and to extend over longer distances in WL compared to BL lines. This can be attributed to the very effective and intense long-term selection in the WL population. In contrast, the BL was originally a dual-purpose breed selected for multiple objectives and was subject to advanced selection procedures much later than WL. Furthermore, white egg layers originate from a single breed only, the single comb White Leghorn, while the genetic base of brown layers was broader [38]. A first study of the level of LD in chicken was conducted on a number of breeding populations of layer chickens [8]. In this study LD was evaluated using the standardized chi-square (χ^2^) measure and showed appreciable LD among markers up to 5 cM apart. This study, however, was based on microsatellites instead of SNPs and uses a different statistic compared to our study. Aerts et al. [33] investigated the extent of LD on the two chromosomes 10 and 28 in a white layer population and two broiler chicken breeds. The inbred layer breed showed an extent of useful LD up to 4 cM on chromosome 10. They also found that the extent of LD varies dramatically. In a study of Andreescu et al. [9] genotype data for 959 and 398 SNPs on chromosomes 1 and 4 from nine commercial broiler chicken breeding lines were used. They reported that *r^2 ^*extends only over relatively short distances (< 1 cM). Further studies with 3 K SNP data suggested that in general the level of LD in WL (*r*^2 ^> 0.2 at < 2 Mb) is higher than broilers [11]. While the 3 k chip was not of sufficient density to reveal LD statistics, it was adequate for studying impacts of commercialization on allelic diversity and inbreeding. A study by Muir et al. [32] reported a higher level of inbreeding and lower allelic diversity in broilers than in egg laying chickens which is consistent with the results of this study.

In the current study, we aimed at dissecting the LD pattern more precisely by employing a higher marker density. Our results suggest that the level of LD in brown egg laying chickens is much less than previously assumed. However, the level of LD in the white egg layer lines we studied appears to be similar to the values reported with a lower marker density, which is consistent with the relatively small estimated effective population sizes of WL populations.

The decay of LD in a genome determines the resolution of quantitative trait loci detection in association mapping studies and indicates the required marker density. The substantial differences in the LD pattern between lines reflected by different relationship between *r^2 ^*and the physical chromosome length (Figure 4) confirms the complex nature of LD in chicken. This has consequences for QTL mapping and, in particular, fine mapping studies. LD in macrochromosomes extends over large genomic regions so that there is a higher chance of finding association between a gene affecting a particular phenotype and a marker at a given distance. This means that a modest number of markers should be sufficient to identify macrochromosomal QTL by association mapping. However, with long-range LD, the physical distance between a gene and an associated marker can be substantial, making fine mapping and gene identification more challenging. Conversely, on microchromosomes, the fast decay of LD over short distances requires a much denser marker map for finding associations. However, when found, gene annotation and identification will be less tedious [39].

For genomes of mammalian farm animals (e.g. cattle) with an approximate average ratio of 1 Mbp/1 cM, the required level of LD (*r*^2^) for genomic association studies was suggested to be in range of 0.2-0.3 [34,23]. Considering that the linkage distance in chicken is on average 3 times larger than in mammals, current results indicate that the SNP spacing should be ~100 Kb and ~35 Kb, respectively, for commercial white and brown layers to achieve a comparable coverage in future population wide studies with a whole-genome approach. This implies that the current density of informative SNPs used may be adequate, provided that SNPs are distributed across the micro- and macrochromosomes proportionally to their recombination rates.

The extent of haploblock structures in commercial populations of chicken is not well enough documented to allow a comparison with the results of the current study. Most recently, Megens *et al. *[40] reported haploblock length of less than 10 kb in the genome of some commercial lines. However, the study was limited to targeted regions of only two macro and two micro-chromosomes covered by SNP sets of high density. In contrast to the haploblock structure in other species, average block size observed in the present study is comparable to the one recently observed in cattle [17] and at the same time is 20-30 times larger than the one reported in human genomes [41]. It must be noted that the marker density used in this study is more than 50 times sparser than the one currently being used for the human genome. In cattle only a minor part (i.e., 4%) of the genome could be assigned to haplotype blocks, indicating that the SNP density was not sufficient. With a denser bovine SNP panel, shorter blocks will be identifiable and the average haploblock size is expected to decrease. In the current study, the haploblocks account for a substantial proportion of the entire genome coverage (especially so in the white layers), hence a denser SNP panel will not lead to the identification of large numbers of short blocks, but in agreement with the observations by Megens *et al. *[40] the large blocks found with a coarse SNP panel may possibly split up in series of small blocks with a denser panel.

In typical livestock breeding populations, where intensive selective breeding is practiced, effective population size plays a central role because it affects both the degree to which a population can respond to selection and its sensitivity to inbreeding effects [42-44]. Given these implications, the knowledge of *N*_e _facilitates the design of efficient artificial selection schemes [45]. As highlighted by Wang [46]*Ne *is notoriously difficult to estimate, mainly due to the highly stochastic nature of the processes underlying inbreeding and genetic drift.

**Figure 7:**
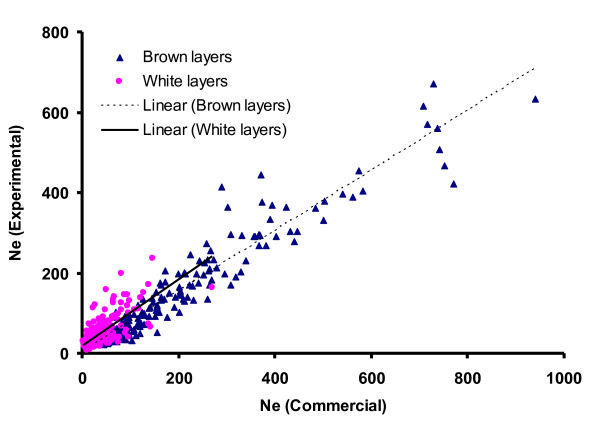
**Estimates of *Ne *values from experimental white and brown layers are plotted against the commercial lines**. *R^2 ^*of fitted lines were estimated as 0.92 and 0.57 for white and brown layers, respectively.

We estimated historical *Ne *based on information from LD for each population separately. The results were found to be consistent between analyses based on the parallel SNP chips, indicating that results were independent of the panel used, as expected when SNPs are neutral and most LD is generated by drift (Figure 7).

The effective population sizes that were estimated in experimental lines are generally in agreement with current estimates of *Ne *based on information from pedigrees of these populations [37]. Our estimates of *Ne *in layer lines are substantially smaller than those obtained for the broiler lines reported by Andreescu et al. [9], which confirms the higher estimates for broiler than for layer lines observed by Muir et al. [32]. Indeed, differences in *Ne *between WL and BL lines are consistent with the breed histories of those lines.

## Conclusions

This is a comprehensive study of LD based on high density SNP panels in breeding lines of egg laying chickens and currently reflects the best estimates of genome-wide LD based on the number of animals screened and number of SNPs genotyped. The number of usable SNPs in this study was 19 k to 37 k in brown layers and 8 k to 19 k in white layers. The results were found to be consistent between analyses based on the parallel SNP chips and across different types of populations (commercial vs. experimental) within the brown and the white layers, respectively. We found that LD generally decays rapidly with distance in different lines, but there was substantial variation between WL versus BL and subtle difference between commercial and experimental populations. If the pattern observed in this study holds true for most of the genome, it is indicated that the current density of usable markers in this study is sufficient for association mapping and implementation of genomic selection within these populations to achieve a comparable resolution and accuracy as in mammalian farm animal populations with applications of 50 to 60 k SNP chips. If genomic predictions are to be made across lines, higher SNP densities may be advantageous.

## List of abbreviations

LD: Linkage disequilibrium; Ne: effective population size; WC: white commercial; WE: white experimental; BC: brown commercial and BE: brown experimental

## Authors' contributions

SQ performed the statistical analyses and wrote the final draft and prepared the manuscript for submission. HS supervised the study and contributed in the conception of the study and revising and editing the manuscript. MH collaborated in generation of data. SW participated in provision of data and in reviewing the manuscript for scientific content. RP participated in provision of study material and manuscript improvement and also provided administrative support. All authors read and approved the manuscript.
